# Contrasting Effect of Prepulse Signals on Performance of *Toxoplasma*-Infected and *Toxoplasma*-Free Subjects in an Acoustic Reaction Times Test

**DOI:** 10.1371/journal.pone.0112771

**Published:** 2014-11-10

**Authors:** Lenka Příplatová, Blanka Šebánková, Jaroslav Flegr

**Affiliations:** Faculty of Science, Charles University, Dept. Philosophy and History of Science, Prague, Czech Republic; NIH, United States of America

## Abstract

**Background:**

About 30% of people on Earth have latent toxoplasmosis. Infected subjects do not express any clinical symptoms, however, they carry dormant stages of parasite *Toxoplasma* for the rest of their life. This form of toxoplasmosis is mostly considered harmless, however, recent studies showed its specific effects on physiology, behaviour and its associations with various diseases, including psychiatric disorders such as schizophrenia. Individuals who suffer from schizophrenia have about 2.7 times higher prevalence of *Toxoplasma*-seropositivity than controls, which suggests that some traits characteristic of schizophrenic patients, including the sex difference in schizophrenia onset, decrease of grey matter density in specific brain areas and modification of prepulse inhibition of startle reaction could in fact be caused by toxoplasmosis for those patients who are *Toxoplasma*-seropositive.

**Methodology/Principal Findings:**

We measured the effect of prepulse inhibition/facilitation of the startle reaction on reaction times. The students, 170 women and 66 men, were asked to react as quickly as possible to a startling acoustic signal by pressing a computer mouse button. Some of the startling signals were without the prepulse, some were 20 msec. preceded by a short (20 msec.) prepulse signal of lower intensity. *Toxoplasma*-seropositive subjects had longer reaction times than the controls. Acoustic prepulse shorted the reaction times in all subjects. This effect of prepulse on reaction times was stronger in male subjects and increased with the duration of infection, suggesting that it represented a cumulative effect of latent toxoplasmosis, rather than a fading out after effect of past acute toxoplasmosis.

**Conclusions:**

Different sensitivity of *Toxoplasma*-seropositive and *Toxoplasma*-seronegative subjects on effect of prepulses on reaction times (the toxoplasmosis-prepulse interaction) suggested, but of course did not prove, that the alternations of prepulse inhibition of startle reaction observed in schizophrenia patients probably joined the list of schizophrenia symptoms that are in fact caused by latent toxoplasmosis.

## Introduction

A protozoan parasite *Toxoplasma gondii* infects about 30% of inhabitants of both developed and developing countries [1]. Since 1994, more and more studies have shown that in the latent phase of *Toxoplasma* infection, which was previously considered asymptomatic from a medical point of view, specific changes are induced in behaviour and the physiology of infected humans and of artificially infected animals [2], [3]. While some of the observed changes are considered the results of manipulative activity of the parasite aimed to increase the chance of transmission of parasites from intermediate hosts (any warm-blooded animal) to the definitive host of *Toxoplasma*, the cat [2]–[4], others are probably just non-specific side effects of *Toxoplasma* infection on the host physiology, e.g. of mild but long term impairment of health or of a mild chronic stress [5].

Studies of *Toxoplasma*-induced changes performed in the previous decades showed personality shifts [6],[7], changes in simple reaction times and intelligence [8], [9], and also suggested possible association between toxoplasmosis and several psychiatric diseases, especially schizophrenia [10]–[13]. Results of recent metastudies analysing, around 50 studies, showed schizophrenia sufferers to have a, approximately, 2.7 times higher probability of being *Toxoplasma* infected than healthy controls [14]–[16]. Recent studies showed that at least some known effects of schizophrenia occur only in schizophrenia patients infected with *Toxoplasma,* with a frequently reported difference in an onset of schizophrenia in men and women [17] and a change in grey matter density observed in schizophrenia patients [18] being the most prominent examples. These observations as well as, for example, more serious positive clinical symptoms of schizophrenia suggest that there are at least two types of schizophrenia prevalent in the population with the most serious one associated with toxoplasmosis [12], [17], [19] as well as that some of the reported differences between schizophrenics and normal controls can be in fact caused by toxoplasmosis itself rather than by schizophrenia. This can be true for the observed sex differences in the onset of schizophrenia, but not the decrease in density of grey matter as it was observed only in *Toxoplasma*-infected patients but not in *Toxoplasma*-infected controls [20]. Other traits that are considered to be characteristic of schizophrenia patients but could be potentially characteristic for *Toxoplasma* infected subjects are olfactory changes and differences in prepulse inhibition of startle reaction (PPI). Specific changes in olfactory functions and preferences were observed both in schizophrenia patients [21], [22] and in *Toxoplasma* infected healthy humans [23] and animals [13], [24]–[27]. Similarly, the effect of schizophrenia on startle reaction in prepulse inhibition test is widely known and is even suggested to be a sensitive tool for research of various types of mental disorders and neurobiological problems [28] including schizophrenia [29], [30]. Male schizophrenic patients with early onset of the disease were reported to have profound deficits in PPI of startle response [31]. Modulation of startle response was also found in people with schizotypal personality disorder and even in relatives of schizophrenic patients [32], suggesting possibility to find PPI deficits also in healthy subjects with predispositions to schizophrenia. A recent study showed, that not only schizophrenia patients [33], but also *Toxoplasma*-infected controls have increased latency of startle reflex and decreased effect of prepulse on the latency than *Toxoplasma*-free subjects in acoustic startle reflex inhibition test [34].

While the studies suggesting an association between the latent toxoplasmosis and schizophrenia seem convincing, little is known about the mechanism underlying the connection, with the main suspect being the neurotransmitter dopamine, which plays an important role in aetiology and clinical picture of schizophrenia [35], [36]. Research from three main areas back up our understanding that dopamine is also responsible for at least some of the changes associated with latent toxoplasmosis: (1) Genetic research showed *T. gondii* to have two genes encoding thyroxine hydroxylase, a rate-limiting enzyme in dopamine synthesis [37]. (2) Animal research showed changes in dopamine levels in infected mice and in brain tissues [38], [39], as well as dopamine role in observed behavioural changes [40], [41] and in production of *T. gondii* tachyzoites in infected tissues [42]. (3) In humans, behavioural changes associated with latent toxoplasmosis suggest differences in dopamine levels in seropositive subjects [43], [44], and research of human neurotransmitter and neuropeptide systems showed (among other alterations) significant changes in protein levels of DRD1 in cells infected with type I strain of *T. gondii* [45]; DRD1 is involved in negative feedback regulation of dopamine release in the brain [46].

Following our previous studies of simple reaction times [8], we decided to further our understanding of *Toxoplasma*-induced changes in reaction times and sensorimotor gating using an experiment that combines simple reaction times tests with tests of PPI. In experiments conducted with healthy individuals it was shown that a startle signal preceding a go signal decreases reaction time [47]. It would be extremely adaptive for any predation-transmitted parasite to decrease or even switch off this startle facilitated reaction of an infected host, so called startReact [47], [48], by, for example, decreasing the intensity of its startle reaction. It is known that PPI plays a significant role in modifying the startle while not affecting the decrease in reaction times [49]. However, in the original study the go signals and startle signals were different and of a different modality, namely using optical go signals and acoustic startle signals. Under natural condition, when a feline predator attracts its prey, the same signal usually plays a role of both go and startle signal. Therefore, we modified the usual setup of startReact experiments by using the startle acoustic signal as the go signal. Using this more ecologically relevant setup we tested the hypothesis that modulation of startle reaction by prepulses could influence reaction times of *Toxoplasma*-infected and *Toxoplasma*-free subjects differently.

## Materials and Methods

### Ethics statement

The study was approved by IRB of Faculty of Science (Etická komise pro práci s lidmi a lidským materiálem Přírodovědecké Fakulty Univerzity Karlovy) and all experimental subjects signed the informed consent before the start of the study.

### Experimental subjects

236 biology students of Charles University in Prague (170 women and 66 men, the ratio corresponding to the sex ratio of Faculty of Science students’ population) agreed with participating in the double-blind study. All subjects were Caucasians of a Czech or Slovak (<10%) nationality. A socioeconomic stratification of population of past Czechoslovakia is very low and this is especially true for population of students of Charles University, the most prestigious Czech university. No student reported any hearing problem and visual inspection of data suggested that no subject with such problems was among participants of reaction times test. All students underwent blood sampling for serological analysis, battery of psychological tests (results not used in the present study) and PC test of acoustic prepulse inhibition of simple reaction times. During the experiment, neither the subjects nor the laboratory assistant were aware of results of serological tests for toxoplasmosis. The students were paid 400 CZK (equivalent of $ 20) for their participation in the study.

### Serological analysis

Blood samples (1 ml of frozen sera) obtained from participants were sent to National Laboratory for Toxoplasmosis, National Institute of Public Health, Prague and screened for specific anti*-Toxoplasma*_IgG antibodies using ELISA (SEVAC, Prague, Czech Republic) and complement fixation test (CFT, SEVAC, Prague, Czech Republic). Samples with high titres of IgG antibodies were tested for IgM (TestLine, Brno, Czech Republic) to exclude possible cases of acute toxoplasmosis. CFT titres from 1∶8 to 1∶128 are assumed to signify latent *T. gondii* infection [50]. CFT results rather than results established by ELISA were used as CFT is more reliable in case of longer duration *Toxoplasma* infection [50]. Subjects with CFT titres 1∶8 and higher and in the same time positive in the ELISA test (positivity index higher than 1.0) were considered *Toxoplasma*-infected, the four subjects with opposite results of CFT and ELISA tests as well as one subject positive in IgM tests were excluded from the study.

### Psychomotor test

A PC test of acoustic prepulse inhibition of simple reaction times was developed on our workplace based on previously used test of simple reaction times [51]. The Windows XP, 7, 8 compatible program is available at http://web.natur.cuni.cz/flegr/programy.php.

During the test, a plain grey screen with one button in the middle was presented and the subject was asked to click the left mouse button each time the sound stimuli was played. Bursts of white noise in intensity and duration used in standard tests of PPI were used in our experiment (duration of prepulse signal 20 ms, duration of startle stimuli 40 ms, background white noise present during the whole experiment), the time interval between prepulse and main stimuli was 20 ms. To avoid reactions to the prepulse instead of the main stimuli, pseudorandom prepulse signals without following stimulus were presented through the test. Stimuli were presented through headphones (Philips SHP1900) with volume set on maximum. All the subjects were tested using identical sets hardware. Three trial signals were played for the experimental subject to get accustomed to the experimental setting before the measured part of the experiment started. 32 prepulse preceded stimuli and 28 plain stimuli were presented in pseudorandom order ensuring similar representation of plain and prepulse preceded stimuli in the first, middle and last part of the program run. The same pseudorandom sequence of signals was used for all subjects. Program ended after the experimental subject responded to the last stimulus. Time from the beginning of the experiment to the last presented stimulus was 4 minutes 17 seconds. Based on a preexperimental testing, the intensity of background noise, prepulses, stimuli and intervals between prepulse and pulse were set to 57 dB, 67 dB, 107 dB and 20 msec., respectively. Under these conditions, different reactions to stimuli with and without prepulses were observed and no reactions of subjects to sole prepulses were observed. The intensity of acoustic signals were measured with calibrated digital storage oscilloscope Agilent DSO 5054A and the acoustic files were edited using Audacity 1.3 software.

### Data analysis

Data were manually filtered to exclude all items longer than 1000 ms or shorter than 100 ms (<10% of data). The mean reaction times for signals with (S_p_) and without (S_n_) prepulse and normalized differences of signals with and without prepulse were computed as Dn = (S_n_–S_p_)/S_n_. The program package Statistica v. 9.0 was used for logistic regression, repeated measure GLM analysis, descriptive statistics and for testing the parametric statistical tests presumptions. For performing the nonparametric partial Kendall regression tests [52] we used Kendall Taus computed with the Statistica and an Excel sheet [53] available at http://web.natur.cuni.cz/flegr/programy.php. Data file for possible reanalysis is available at http://web.natur.cuni.cz/flegr/data/audioPrepulse.txt.

## Results

The final set contained 236 subjects, 170 women (age 21.8, range 19–30, S.D. 2.12) and 66 men (age 22.2, range 19–31, S.D. 2.74). Forty four (44) *Toxoplasma* infected subjects were older than 192 *Toxoplasma*-free subjects (22.7 vs 21.7, t_234_ = 2.60, p = 0.01). Logistic regression with sex and age as independent factors showed no effect of sex (O.R. 0.86, C.I._95_ = 0.42–1.79, p = 0.69) and positive effect of age (O.R._95_ 0.142 (range), C.I._ 95_ = 0.03–0.68, p = 0.015) on probability of being *Toxoplasma*-infected.

Students missed less than 2% of stimuli and less than 10% of reaction times were too short (less than 100 msec. or too long (more than 1000 msec.). These suspect data were eliminated manually before the mean reaction times were calculated. No reactions on standalone prepulse signals were registered.

**Figure 1:**
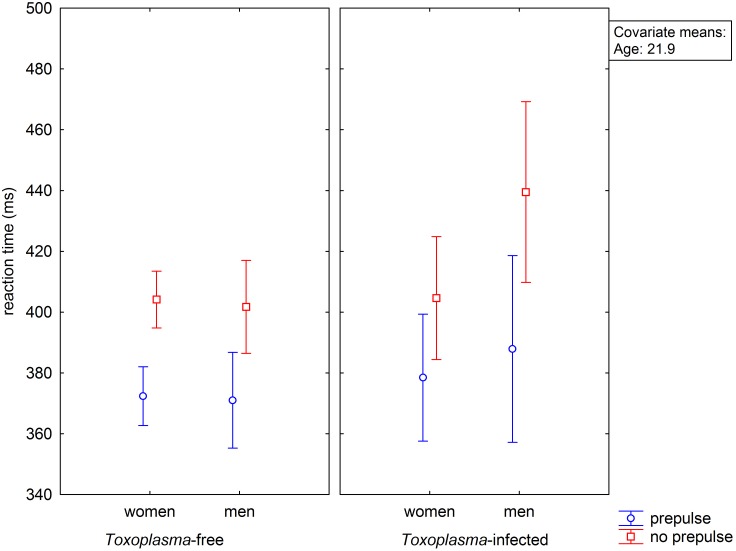
Influence of toxoplasmosis, prepulse and sex on reaction time. Vertical bars denote 0.95 confidence intervals.

**Figure 2:**
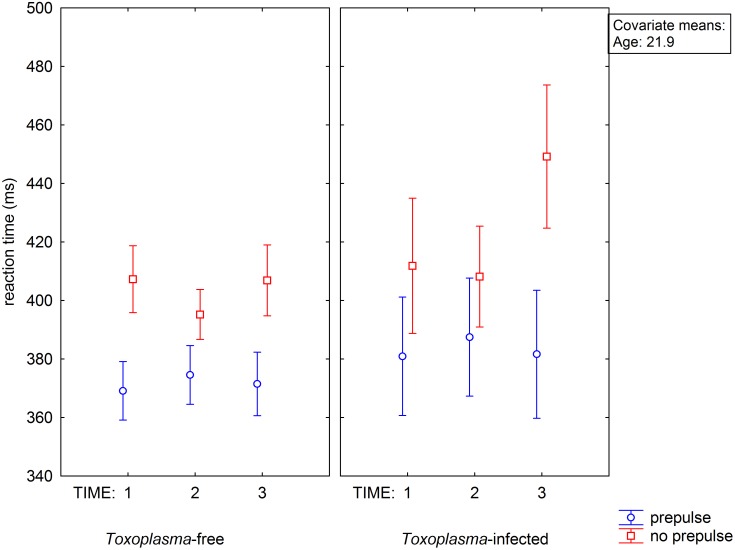
Influence of toxoplasmosis and prepulse on reaction time in different phase of experiment. Vertical bars denote 0.95 confidence intervals.

Repeated measure GLM with two dependent variables, namely mean reaction time with and without prepulse and independent variables toxoplasmosis (meaning seropositive subjects with latent infection), sex and age showed no main effect of age (p = 0.77, µ^2^<0.001), toxoplasmosis (p = 0.128, µ^2^ = 0.010), sex (p = 0.308, µ^2^ = 0.004) and prepulse (p = 0.262, µ^2^ = 0.005) but significant effect of prepulse-sex (p = 0.035, µ^2^ = 0.020) and prepulse-toxoplasmosis-sex (p = 0.020, µ^2^ = 0.023) interactions, see Fig. 1. The presence of prepulse was associated with shorter reaction times and this effect (prepulse facilitation) was especially strong in *Toxoplasma*-infected men. Since our previous results suggested that toxoplasmosis negatively influences the ability of long-term concentration, rather than reaction times under ideal conditions of maximum concentration at the start of reaction time-tests [8], [54], we repeated the GLM analysis with six dependent variables – the mean reaction time with and without prepulse for the 1^st^, 2^nd^, and 3^rd^ third of the test, and independent variables toxoplasmosis, sex and age. The results of the test suggested the effects of time-toxoplasmosis-prepulse interaction (p = 0.010, µ^2^ = 0.020), time-toxoplasmosis-sex (p = 0.013, µ^2^ = 0.019), time-prepulse-sex (p = 0.026, µ^2^ = 0.016) but not time-toxoplasmosis-prepulse-sex (p = 0.095, µ^2^ = 0.010). The separate GLM analyses for three parts of the tests showed that the effects of toxoplasmosis (p = 0.026, µ^2^ = 0.022), toxoplasmosis-sex interaction (p = 0.028, µ^2^ = 0.021), prepulse-toxoplasmosis interaction (p = 0.009, µ^2^ = 0.029), prepulse-sex interaction (p = 0.006, µ^2^ = 0.031) and prepulse-toxoplasmosis-sex interaction (p = 0.014, µ^2^ = 0.026), were significant only for the final part of the test (Fig. 2).

**Figure 3:**
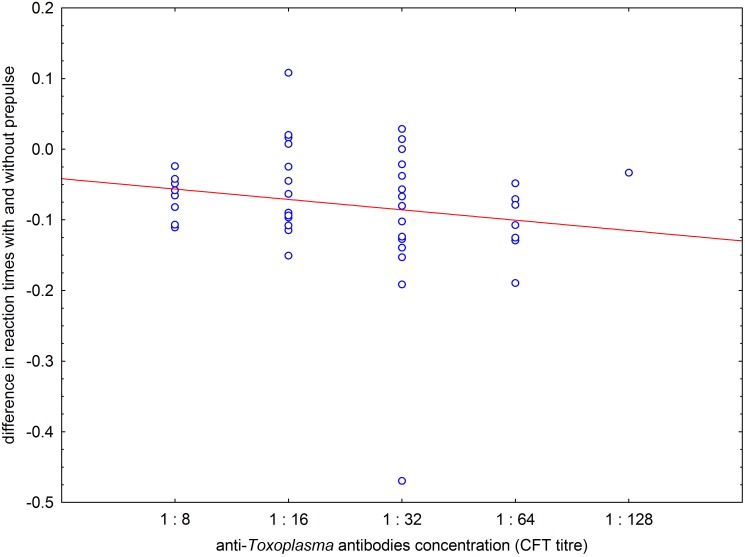
Correlation of anti-*Toxoplasma* antibodies titres with effect of prepulse on reaction times in *Toxoplasma*-infected subjects. Effects of prepulse on reaction time of each rater was calculated as the difference of his mean reaction times with and without prepulse/his mean reaction times without prepulse.

To reveal whether observed statistical association represents the cumulative effect of latent toxoplasmosis or rather only a vanishing after-effect of past acute toxoplasmosis, we searched for a possible association between the concentration of specific anti-*Toxoplasma* antibodies (namely CFT titre) and the normalised difference between mean reaction time with and without prepulse. The age of subjects was controlled by using partial Kendall correlation test with ordinal variable CFT titre as a factor and continuous variable age as a covariate. In 44 *Toxoplasma*-positive subjects, the difference between reaction time with and without prepulse correlated negatively with concentration of anti-*Toxoplasma* antibodies (p = 0.025, Tau = −0.201, one-sided test), Fig. 3. Statistically, concentration of specific anti-*Toxoplasma* antibodies decreases with duration of latent toxoplasmosis. Therefore, existence of a negative correlation between difference in reaction times with and without prepulse suggested that the observed toxoplasmosis-prepulse interaction increased with time from the moment of *Toxoplasma* infection.

## Discussion

Results of our study performed on 236 students showed that the *Toxoplasma* infected subjects, especially men, had significantly prolonged reaction times to simple acoustical signals. All subjects reacted more quickly on acoustical signals that were preceded with a weak prepulse and this positive effect of the prepulse was strongest for *Toxoplasma*-infected men. The observed effects of prepulse-toxoplasmosis interaction increased with duration of *Toxoplasma*-seropositivity estimated on the basis of anti-*Toxoplasma* antibodies concentration, suggesting that the effect is most probably the results of slow and cumulative changes induced by latent toxoplasmosis, rather than of the transient after-effect of past acute toxoplasmosis.

Effect of latent toxoplasmosis on simple acoustical-signal reaction times was stronger in the third part of the test. This agrees with already published studies showing that the difference in reaction times between *Toxoplasma*-seropositive and *Toxoplasma*-seronegative subjects was absent in the first minute of the 3-minutes test, increased to its maximum in the second minute of the test when the performance of *Toxoplasma*-seropositive subjects declined, and decreased again in the third minute of the test when the psychomotor performance of *Toxoplasma*-seronegative subjects declined too [8], [51], [54]. All previous studies showing the effect of latent toxoplasmosis on reaction times used visual rather than acoustical signals without any prepulse. Therefore, the present results, namely the decrease of the effect of latent *Toxoplasma* infection on reaction time on prepulse preceded-signals should be confirmed in future studies using visual signal reaction time tests.

Our study revealed very strong facilitating effects of prepulses on the reaction time in all categories of subjects. The difference was about 30 ms for *Toxoplasma*-free and about 70 ms for *Toxoplasma*-infected men. This prepulse-associated decrease of reaction time cannot be explained by students’ reacting on prepulse rather than on signals as about same number of prepulses was presented alone, without following signal and the students never responded to the standalone prepulse signals. Moreover, the decrease of latency by 70 ms in *Toxoplasma* infected subjects was larger than the interval between pulse and prepulse. Similar but weaker effect of acoustical prepulses on startle reflex latency (not on the simple reaction time) was already reported in [34]. The most probable explanation of the effect observed in our study is that the prepulse either decreases possible startle response of probands triggered by signals that could otherwise prolong reaction times of all subjects [55], or conveys attention of probands to the following signal.

Our *a priory* hypothesis was based on the former hypothesis, however, the obtained data are in better agreement with the latter one. In the questionnaire studies, the *Toxoplasma*-infected subjects answer that they have no or weak and slow startle reaction [7]. We expected that due to their weaker startle reaction, reaction times of *Toxoplasma*-infected subjects will be less affected by prepulse than the *Toxoplasma*-free controls. However, our results suggest the opposite – the positive effect of prepulse was stronger in the *Toxoplasma*-infected subjects than in the *Toxoplasma*-free subjects. It must be acknowledged, however, that the effects of prepulse on startle reaction, both on its intensity and latency, are highly context dependent and, for example, for many combinations of intensity and signal-prepulse interval the inhibition can turn into the enhancement [49], [56], [57]. The most parsimonious, but still speculative, explanation of the present phenomenon is that the *Toxoplasma*-infected subjects, especially those that are infected for very long time, lose their concentration during the third part of the reaction time test and the prepulse conveys their “attention” to immediately succeeding signal. It must, however, be emphasized that the experimental subjects did not recognized the existence of two types of signals, the signals with and without the prepulse. Their reactions to the prepulses were therefore purely subconsciously driven by some extra cortical circuit.

The results of present studies supported, but of course not proved, our notion suggesting that many reported effects of schizophrenia, including the aberrant effects of prepulses in the startle inhibition tests, are in fact effects of *Toxoplasma* infection due to increased prevalence of subjects with latent toxoplasmosis in schizophrenia patients [14]–[16]. However, the study of Pearce et al. [34] showed that schizophrenia patients had larger startle reflex latency than the controls regardless of their toxoplasmosis-status, i.e. no significant schizophrenia-toxoplasmosis interaction was observed in their study. Similarly, they found no effect of schizophrenia-toxoplasmosis-prepulse or toxoplasmosis-prepulse interactions. This seems to contradict our results showing toxoplasmosis-prepulse interaction and also to our hypothesis according to which *Toxoplasma*-free schizophrenia patients are expected to have similar effect of prepulses on startle reflexes as *Toxoplasma*-free controls. It must be reminded, however, that startle reaction, which has the latency about 50 ms after the “go” signal is very different process than oriented reaction in simple reaction time tests. Startle reaction is reliably modified by cognitive and emotional processes [56] but the same is even more true for far more complex oriented reaction. Both studies included similar number of *Toxoplasma*-infected subjects, however, by analysing differences of mean with- and without-prepulse reaction time calculated separately for each subject we used more sensitive within-subject technique in searching for toxoplasmosis-prepulse interaction. It must be also reminded that that the commercial diagnostic kits are optimized for diagnosis of acute and postacute toxoplasmosis and have relatively high frequency of false negative results in subjects with old parasite infection and therefore with low concentration of antibodies [58]. Moreover, recent results show that the diagnostic kits have too high specificity, they probably detect just the most common strains of *Toxoplasma gondii* [59]. This can be solved either by using several different tests based on different antigens, which is the only possibility in the clinical practice, or by analyzing the data with special permutation tests for false negative results contaminated-data, rather than with standard statistical test [60], which is the solution applicable in the research project only. While most of latent toxoplasmosis researchers including Pearce et al. [34] use just one diagnostic test for the diagnosis of the *Toxoplasma* infection, we always use two tests and exclude about 10% of subjects with discordant results of tests. The possible presence of a larger subpopulation of false negative subjects with oldest infections and therefore also probably with largest cumulative effect of latent toxoplasmosis in subpopulation of *Toxoplasma*-negative patients than in *Toxoplasma*-negative controls could explain observed effects of prepulses on startle reaction in *Toxoplasma*-negative schizophrenia patients.

### Limitations of present study

The effect of toxoplasmosis-prepulse interaction was stronger in male students. In the same time, the number of men in the Faculty of Science biology students and therefore also in our experimental set was rather low – only 14 male students were *Toxoplasma*-infected. It will be therefore necessary to repeat a similar study with a larger sample. Such repetition is very urgent because the effect of *Toxoplasma*-seropositivity is known to depend on RhD phenotype and genotype of subjects [61], [62]. The negative effects of *Toxoplasma*-seropositivity are usually much stronger in RhD negative subjects and are sometimes even absent in the most numerous RhD positive heterozygotes [61]. However, the number of RhD negative subjects is about 16% in the Czech population and therefore much larger experimental sample will be necessary to collect for controlling this important confounding factor. It must be noted, however, that the RhD negative subjects were equally represented in *Toxoplasma*-infected and *Toxoplasma*-free men (they were no significantly more frequent in *Toxoplasma*-infected than *Toxoplasma*-free women) and that an uncontrolled but balanced confounding factor increases the risk of false negative but not the false-positive results [2]. *Toxoplasma* is known to influence not only the reaction times but also a level of testosterone and therefore also competitiveness (and motivation) of subjects [63]. Therefore, the effect of *Toxoplasma* infection on performance in particular tests can be even opposite (positive) in different populations [2]. Therefore, our university students-study should be repeated also on different populations such as soldiers or blood donors or on the general population sample. Test subjects in the presented study weren’t tested for hearing inaccuracy; although the tests subjects were mostly healthy young individuals, it is possible several of them might have a minor hearing problem that could affect the presented results.

On the basis of the negative correlation between the effect of prepulse stimuli and concentration of anti-*Toxoplasma* antibodies we suggested that the observed effect of latent toxoplasmosis increases with time passed from the moment of infection. It must be mentioned, however, that the concentration of antibodies only indirectly reflects the time passed from the moment of infection and can also be influenced by other factors, e.g. the intensity of the infection and the immunocompetence of a subject.

We confirmed that latent toxoplasmosis influenced the performance of subjects in psychomotor performance tests. We also confirmed earlier observations indicating that the ability of long-term concentration rather than the best reaction times are influenced by the infection. For the first time we demonstrated different effects of prepulse on reaction times of *Toxoplasma*-infected and *Toxoplasma*-free subjects, especially males. Existence of this effect independently suggests that the ability of concentration most probably plays the role in the latent toxoplasmosis-associated impairment of performance in reaction time tests. In the present paper we studied the effect of prepulse on the reaction times and not on the startle reaction, which latter is the subject of most of prepulse studies, or on StartReact [47], [48], but on latency facilitation of oriented reactions. Our results support the hypothesis that the increased prevalence of toxoplasmosis in schizophrenia patients in connection with atypical responses of *Toxoplasma*-infected subjects to prepulses could be responsible for the well-known differences in reactions of schizophrenia patients in prepulse inhibition of startle reaction tests.
